# Cell membrane-covered nanoparticles as biomaterials

**DOI:** 10.1093/nsr/nwz037

**Published:** 2019-03-14

**Authors:** Mingjun Xuan, Jingxin Shao, Junbai Li

**Affiliations:** Beijing National Laboratory for Molecular Sciences (BNLMS), CAS Key Lab of Colloid, Interface and Chemical Thermodynamics, Institute of Chemistry, Chinese Academy of Sciences (ICCAS), Beijing 100190, China

**Keywords:** cell membranes, synthetic nanosystem, bio-stealth, long circulation time, biomedical applications

## Abstract

Surface engineering of synthetic carriers is an essential and important strategy for drug delivery *in vivo*. However, exogenous properties make synthetic nanosystems invaders that easily trigger the passive immune clearance mechanism, increasing the retention effect caused by the reticuloendothelial systems and bioadhesion, finally leading to low therapeutic efficacy and toxic effects. Recently, a cell membrane cloaking technique has been reported as a novel interfacing approach from the biological/immunological perspective, and has proved useful for improving the performance of synthetic nanocarriers *in vivo*. After cell membrane cloaking, nanoparticles not only acquire the physiochemical properties of natural cell membranes but also inherit unique biological functions due to the presence of membrane-anchored proteins, antigens, and immunological moieties. The derived biological properties and functions, such as immunosuppressive capability, long circulation time, and targeted recognition integrated in synthetic nanosystems, have enhanced their potential in biomedicine in the future. Here, we review the cell membrane-covered nanosystems, highlight their novelty, introduce relevant biomedical applications, and describe the future prospects for the use of this novel biomimetic system constructed from a combination of cell membranes and synthetic nanomaterials.

## INTRODUCTION

Prompted by substantial developments in nanotechnology and biotechnology, revolutionary changes have made crucial contributions to the biomedicine-related areas of drug delivery, diagnostic imaging, and therapeutic approaches [1–7]. Versatile and innovative nanosystems have been specifically designed and developed with a small size, high payload, and flexible functions [8–10]. Meanwhile, several specific ligands, such as peptides, DNA/RNA aptamers, and antibodies, have been conjugated with nanosystems to enhance the targeting recognition and therapeutic efficacy [11–13]. In combination with an additional stimulus, such as a magnetic field, light, and ultrasound, synthetic nanosystems successfully achieve the purposes of efficient delivery and precise therapy [14–17]. Therefore, these advantages have significantly improved the potential of nanosystems for use in biomedical applications.

Although current delivery systems used for *in vivo* applications have received considerable attention, they still suffer from delivery barriers in the presence of immune clearance, biological adhesion, and targeting specific sites [18,19]. Therefore, scientists have devoted their efforts to improving the half-life of synthetic nanosystems and overcoming the bioadhesion in the bloodstream. PEGylation and phospholipid modifications have proved useful for prolonging the circulation time due to their good hydrophilicity, which further inhibits uptake by the reticuloendothelial system (RES) and reduces the bioadhesion of blood components [20]. Despite the successful implementation of PEGylation and phospholipid modifications, synthetic nanosystems that persist in living organisms easily trigger an immune response, followed by rapid elimination by the immune system. Thus, a biointerfacing approach that improves current synthetic nanosystems, achieves immune evasion and prolongs the circulation time must be developed.

Natural cell systems composed of red blood cells, stem cells, and bacteria have been designed as novel carriers for *in vivo* drug delivery [21,22]. However, the microscale size of cell-based delivery vehicles is insufficient to achieve deep tissue penetration and accumulation at the region of interest in living organisms. Based on knowledge from nature, cell membranes are responsible for intercellular communication, immune defence, and metabolism throughout the life circle. Pursuing these smart functions, cell membranes have been extracted by researchers and coated on synthetic nanosystems to prepare cell-like nanosystems from the biomimetic perspective [23,24]. Currently, various types of cell membranes have been extracted as bio-stealth materials to cover 30–300 nm-sized nanoparticles through the processes of physical extrusion, co-incubation or microfluidic fabrication, further improve the *in vivo* performance of nanoparticles and achieve the relevant biomedical applications [25]. Therefore, cell membrane coatings enhance the *in vivo* performance of current nanosystems, which not only display a long circulation time but also combine good biocompatibility and tumour-targeting properties for synthetic nanosystems [26,27].

In this review, we focus on the biohybrid nanosystems that combine natural cell membranes and synthetic nanomaterials. This review will introduce the novel and state-of-the-art of cell membrane-coating technologies and describe a method to extract various cell membranes, such as red blood cells, platelets, immune cells, cancer cells, and *Escherichia coli* (*E. coli*), as the coating materials to mimic synthetic nanosystems. Furthermore, we will explain the membrane-bound functions of cell membrane-covered nanosystems for biomedical solutions and highlight the promising applications to provide a deep understanding of this novel bionic strategy. Cell membrane-covered nanosystems are currently regarded as a novel class of biohybrid materials created using a new method to design and prepare bioinspired theranostic platforms for biomedical applications.

## CLOAKING NANOSYSTEMS WITH CELL MEMBRANES

One of the main purposes of nanomedicine is to achieve efficient delivery and accumulation at the target site *in vivo*. A long circulation time is a necessary feature for active/passive target delivery that should be seriously considered. Surface bioengineering provides a convenient method to improve the circulating performance of nanoparticles *in vivo*. In this case, the cell membrane-coating technology inspires a novel strategy to enable nanoparticle-based delivery systems to freely pass through the bloodstream. Compared with traditional artificial lipid bilayers, natural cell membranes contain a series of cell membrane-related functional moieties (proteins, antigens, and carbohydrates) for functions such as protection, bioantifouling, specific recognition, and intracellular communication. Recently, many types of natural cells, such as red blood cells, platelets, immune cells, cancerous cells, and even *E. coli*, have been developed as the membrane source to construct biohybrid delivery systems with versatile functions through the bottom-up assembly approach.

### Red blood cell membrane-coated nanoparticles

Red blood cells (RBCs) are the most common cell in the blood. As the oxygen delivery carrier, RBCs freely pass through the cardiovascular system and organs without attack from and clearance by the immune system [28–30]. Inspired by this feature, RBC-derived membranes have been designed as stealth coating materials to help nanoparticles evade immune clearance, while improving their circulation lifetime. In the bloodstream, RBCs employ membrane-related immunomodulatory proteins as important moieties for achieving a long circulation time and immune-evasive properties [31–33]. One example is CD47, a transmembrane protein that is responsible for immune evasion; this is capable of inhibiting phagocytosis and anti-inflammatory responses that reduce the immune behaviour of macrophages [27]. As the earliest derived biointerfacing materials composed of cell membrane, the RBC membrane cloaking strategy has been well developed and become a well-known approach to prepare nanoparticle systems with prolonged circulation for biomedical applications ranging from drug delivery to diagnosis and therapy [34]. Although RBC membranes are frequently employed to coat nanoparticles compared with other types of cell membranes, relevant cell membranes will be employed for coating nanoparticles if researchers are pursuing the properties of tissue-specific targeting and immunology.

**Figure 1.**
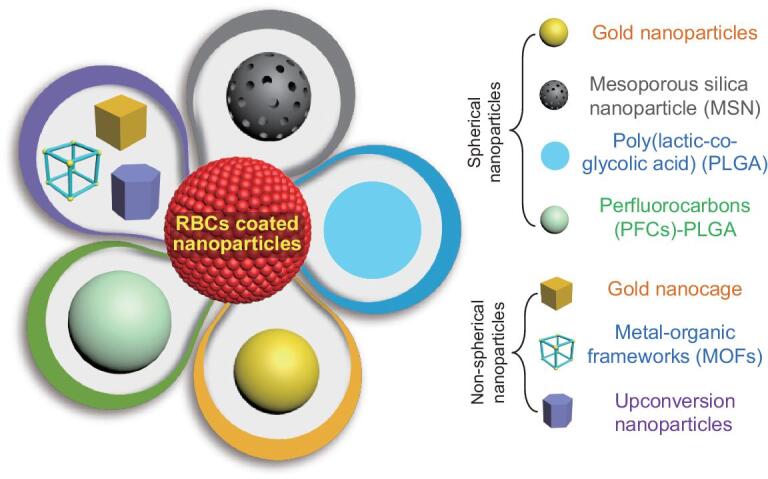
Schematic illustration of different types of RBC membrane-coated synthetic nanoparticles.

**Figure 2.**
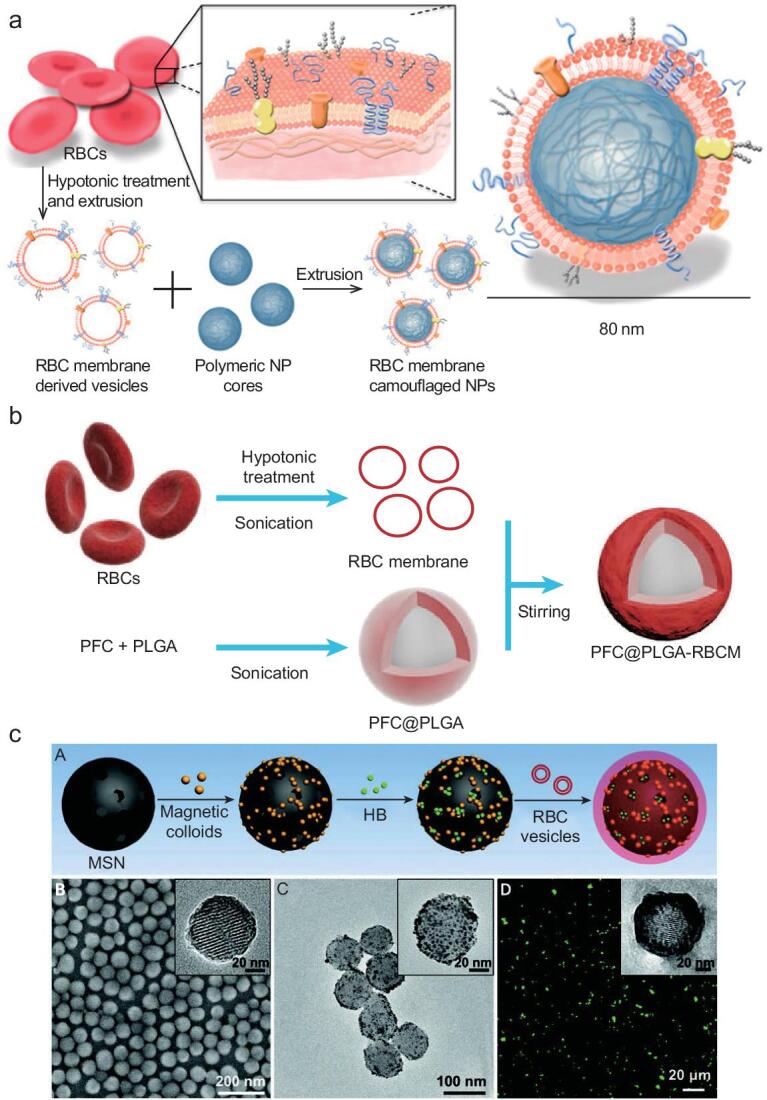
RBC membrane-coated nanoparticles for biomedical purposes: (a) RBC membrane-camouflaged PLGA nanoparticles as a delivery platform with a long circulation time, Copyright 2011, National Academy of Sciences [37]. (b) Erythrocyte membrane-enveloped perfluorocarbon@PLGA nanoparticles for relieving tumour hypoxia and enhancing the efficacy of radiotherapy, Copyright 2017, Wiley-VCH [39]. (c) RBC membrane-coated magnetic mesoporous nanosilica for photodynamic therapy, Copyright 2018, Wiley-VCH [40].

The reconstitution of RBC membranes on the surface of nanoparticles has been achieved using several approaches. According to the classical methods, RBCs are initially emptied to harvest the RBC ghosts by hypotonic treatment [35]. Extrusion and sonication are usually employed to translate the RBC ghosts into nanosized vesicles. After allowing synthetic nanoparticles to mix with RBC vesicles, the reconstitution of RBC membranes occurs on the surface of nanoparticles through mechanically induced physical extrusion, co-incubation, and microfluidic electroporation-facilitated synthesis [36]. Unlike the conventional fabrication of cell membrane-coated nanoparticles, the microfluidic lies on micro-mixing and slice forces for cell membrane-coating process. Using these above approaches, various particles (Fig. 1), such as gold nanoparticles, mesoporous silica nanoparticles (MSNs), poly(lactic-co-glycolic acid) (PLGA) nanoparticles, perfluorocarbons (PFCs)–PLGA nanoparticles, metal–organic frameworks (MOFs), and upconversion nanoparticles, have been used as cores coated with RBC membranes and are capable of long circulation and immunosuppression, radiotherapy, and photodynamic therapy (Fig. 2) [37–40]. With the long circulation time, nanocarriers smoothly cross the bloodstream and aggregate around the tumour tissue of interest, resulting in the improved efficiency of light-sensitive drug delivery and an enhancement of therapeutic efficacy [41–45]. Based on many studies of RBC membrane coatings on synthetic nanoparticles, the coating process does not have strong relationships with the morphology, size, and material type. Some non-spherical particles, such as gold nanocages, MOFs, magnetic clusters, and upconversion nanoparticles, have been successfully coated with RBC membranes for tumour imaging and photothermal therapy [46–49]. Furthermore, RBC membranes have been coated on polymeric nanoparticles and self-propelled motors as agents for blood clearance and antibiotic delivery in response to biological threats caused by a bacterial infection and biological toxins [50,51].

In addition to inheriting the physicochemical properties from RBCs, synthetic nanosystems also conjugate with the RBC membrane coating to form an integrated platform combined with functional guest molecules. Some modularized targeting moieties, such as a folate and nucleolin-targeting aptamer, have been incorporated into the RBC membrane coating through the lipid-insertion approach and subsequently improved the tumour targeting and therapeutic efficacy [52]. Although direct chemical conjugation is not used during the RBC membrane-coating process, RBC membrane-coated particles still maintain their structural stability and functions of the source cells in living organisms. Therefore, based on these derived biological properties, the strategy of RBC membrane camouflage represents a strong and versatile approach for the construction of biomimetic nanovehicles for use in biomedical applications *in vivo*.

### Platelet membrane-coated nanoparticles

Like RBCs, platelets are an essential component of mammalian blood that are responsible for the haemostasis and immune responses, and thus have attracted a great deal of interest from researchers because of their residence in the blood. Platelets have been explored as new coating materials [53,54]. Due to the wide range of related antigens and functional proteins, platelets correlate with immune defence and targeting the injured vasculature, while also responding to invasive microorganisms and playing an important role in tumour metastasis [55–59]. Based on these great properties, platelet membrane-coated nanosystems have been designed and fabricated for a variety of biomedical applications, such as multiple myeloma and thrombus therapy [60], site-specific delivery [54,61], enhanced magnetic resonance imaging [62], targeting and detection of atherosclerosis [63], cancer therapy [64] and the isolation of bacterial threats [65]. Generally, platelet membranes are harvested by gradient centrifugation, and are then coated on the surface of nanoparticles through co-incubation. In addition, a type of hybrid mixed cell membranes derived from RBCs and platelets has been developed as the coating materials for nanosystem to achieve the prolonged circulation time. This hybrid membrane coating brings great opportunities for nanosystem to hold complex biological functions for applications compared with single cell membrane components [66].

### Immune cell membrane-coated nanoparticles

Researchers have continuously studied the residence times of nanoparticles *in vivo* and exploited them for relevant biomedical applications. These findings have further encouraged the development of surface-engineering strategies for nanoparticles through biomimicking approaches. Although RBC membrane-coated particles display great performance in avoiding RES-mediated elimination and immunological surveillance, new types of cell membranes must be developed as the coating material for specific biomedical applications. Researchers pursuing varieties of immunological functions have directed their attention to cells with immune functions, such as macrophages, neutrophils, dendritic cells, stem cells, and T cells, which are capable of mounting an active immune response to promote inflammation, tumour targeting, suppression of tumour metastasis, and virus invasion due to the specific immune functions of cell membrane-bond complex proteins. Thus, strategies employing the specific proteins incorporated in membranes from the aforementioned immune cells to construct biomimetic platforms are very fascinating for researchers.

**Figure 3.**
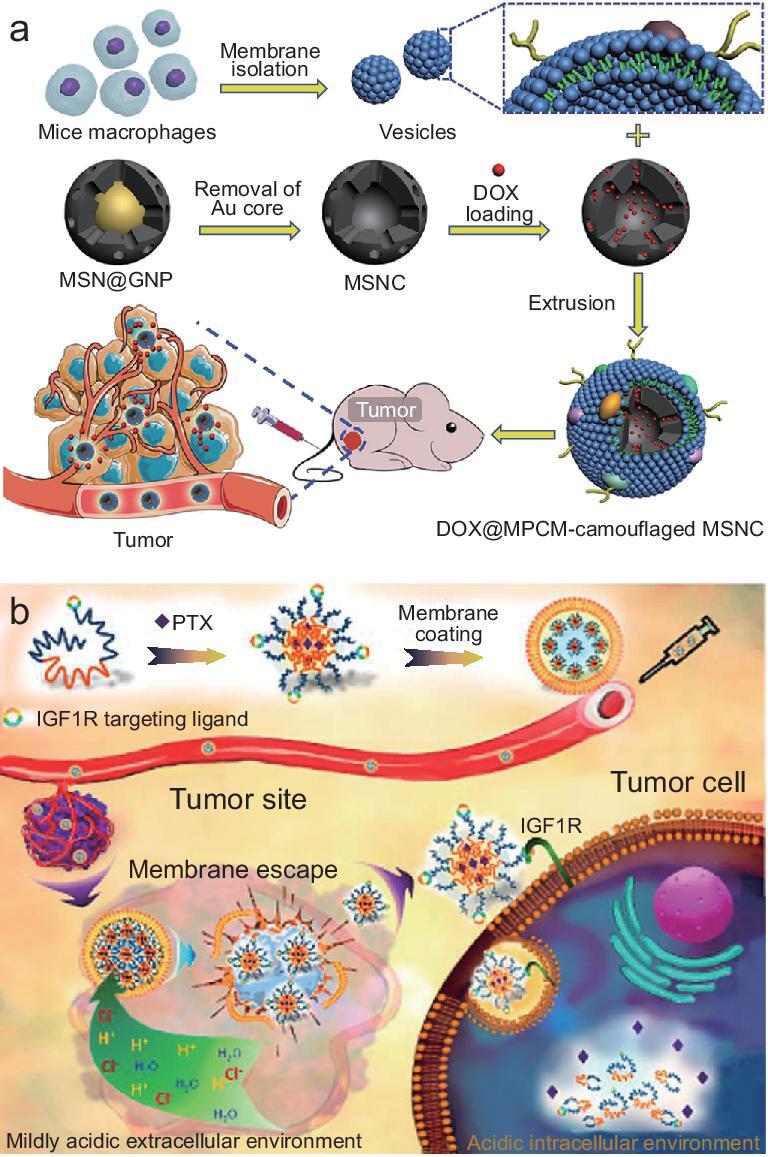
Macrophage membrane-covered nanosystems. (a) Macrophage membrane camouflaged mesoporous silica nanocapsules for DOX delivery and cancer chemotherapy [70], Copyright 2015, Wiley-VCH. (b) Macrophage membrane-coated polymeric nanoparticles for PTX delivery and tumour-targeted chemotherapy [72], Copyright 2018, American Chemical Society.

As ‘lifeguards’, macrophages are immediately activated and recruited to engulf and digest harmful invaders once the signals of infection or tissue damage are detected [67–69]. Based on these fascinating and unique features, macrophage membranes are quite suitable for coating nanoparticles to construct biomimetic hybrid systems for biomedical solutions. Macrophages are emptied through a hypotonic lysis process to acquire the outer membranes. Similar to RBC membrane coatings, the cloaking of the nanoparticle surface with macrophage membrane can be achieved by extrusion or in an ultrasonic bath. Macrophage membrane coatings have proved useful in prolonging the circulation time of silica nanocapsules *in vivo* and their transit through the immune system for anticancer drug (doxorubicin, DOX) delivery, and then further improving the efficiency of drug delivery and therapeutic efficacy (Fig. 3a) [70]. Using the same coating strategy, near-infrared imaging probe (Cy7)-loaded gold nanoshells have been coated with macrophage membrane to achieve increased accumulation in the tumour for bioimaging and enhanced photothermal therapy [71]. Furthermore, a pH-responsive polymeric nanoparticle (cskc-PPiP/PTX@Ma) is coated with macrophage membranes to achieve drug delivery and controlled release by using the cell membrane-coating approach; these nanoparticles are then applied to the tumour-targeted delivery of paclitaxel (PTX) and release in response to the low pH value of endosomes (Fig. 3b). This active drug-delivery cargo combined with cell membranes and pH-responsive polymeric nanoparticles has inspired the rational design of novel *in vivo* delivery systems for the tailored chemotherapy of tumours [72].

Nanoparticles with a regular morphology are usually used as the cores for cell membrane coatings. Researchers have studied the relationship between cell membrane coating and Janus particles, while investigating the therapeutic response in cancer cells. The cell membrane of leukocytes has been employed as a half-coating on the polyelectrolyte part of Au-functionalized Janus polyelectrolyte microcapsules. This Janus capsule uses its half-leukocyte membrane coating to target HeLa cells because of the immune properties of leukocyte membranes [73]. In another example, macrophage cell membranes are half-coated on chemically synthesized Janus mesoporous silica nanomotors through the half protection of PEGlyation and help nanomotors reduce the resistance of biomediums and rapidly adhere to cancer cell membranes for thermomechanically percolating induced therapy [74].

Neutrophils are a type of leukocyte that can migrate through blood vessels. As an essential part of the innate immune system, this ‘unsung hero’ plays an important role in living organisms [75,76]. Activated neutrophils use their cytomembrane function to track cytokines and chemoattractants generated by a wound or inflammation, and accumulate to exert their antiphlogosis effect [77–79]. This chemotaxis behaviour is a highly promising feature to be translated to synthetic nanosystems by coating neutrophil cell membranes on nanoparticles, thereby presenting great potential in drug-delivery systems. The neutrophil cell membrane nanovesicle-based drug-delivery system is created using nitrogen cavitation, which is applied for selectively binding the inflamed vasculature to reverse acute lung inflammation [80]. Meanwhile, a neutrophil membrane-bound protein cocktail has proved useful for capturing circulating cancer cells *in vivo*. The neutrophil membrane-coated PLGA nanoparticles effectively improve the capture efficiency of the circulating cancer cells, specifically enhance homing to the premetastatic niche and inhibit already-formed metastatic lesions [81]. Recently, substantial progress has been achieved in the development of neutrophil cell membrane-coating systems, inspired by the immune response of neutrophils; nanoparticles are coated with neutrophil membranes to construct a platform for inflammatory therapy, which inhibits synovial inflammation and alleviates joint damage in subjects with inflammatory arthritis [82]. Moreover, membranes from a well-known immune cell type, T cells, have also been extracted and employed to coat nanoparticles for the neutralization of HIV infectivity [83].

In addition, we cannot ignore another class of cells in living organisms, stem cells. Stem cells are a special kind of ‘universal cell’ that differentiate into various types of cells to produce the required biological functions. Although stem cells are quite different from defined immune cells, they have the capacity to target tumour cells at different developmental stages due to the hypo-immunogenicity of the cell membrane [84]. Therefore, a series of stem cell membrane-based active drug-delivery systems have been developed for targeted drug delivery and cancer therapy [85]. Accordingly, stem cell membranes are derived and then reconstructed on the surface of gelatin nanogels and upconversion nanoparticles to prolong the circulation time. Meanwhile, stem cell membrane-coated nanoparticles have great potential in tumour-targeted drug delivery and remote-controlled photodynamic therapy *in vivo* [86,87].

### Cancer cell membrane-coated nanoparticles

**Figure 4.**
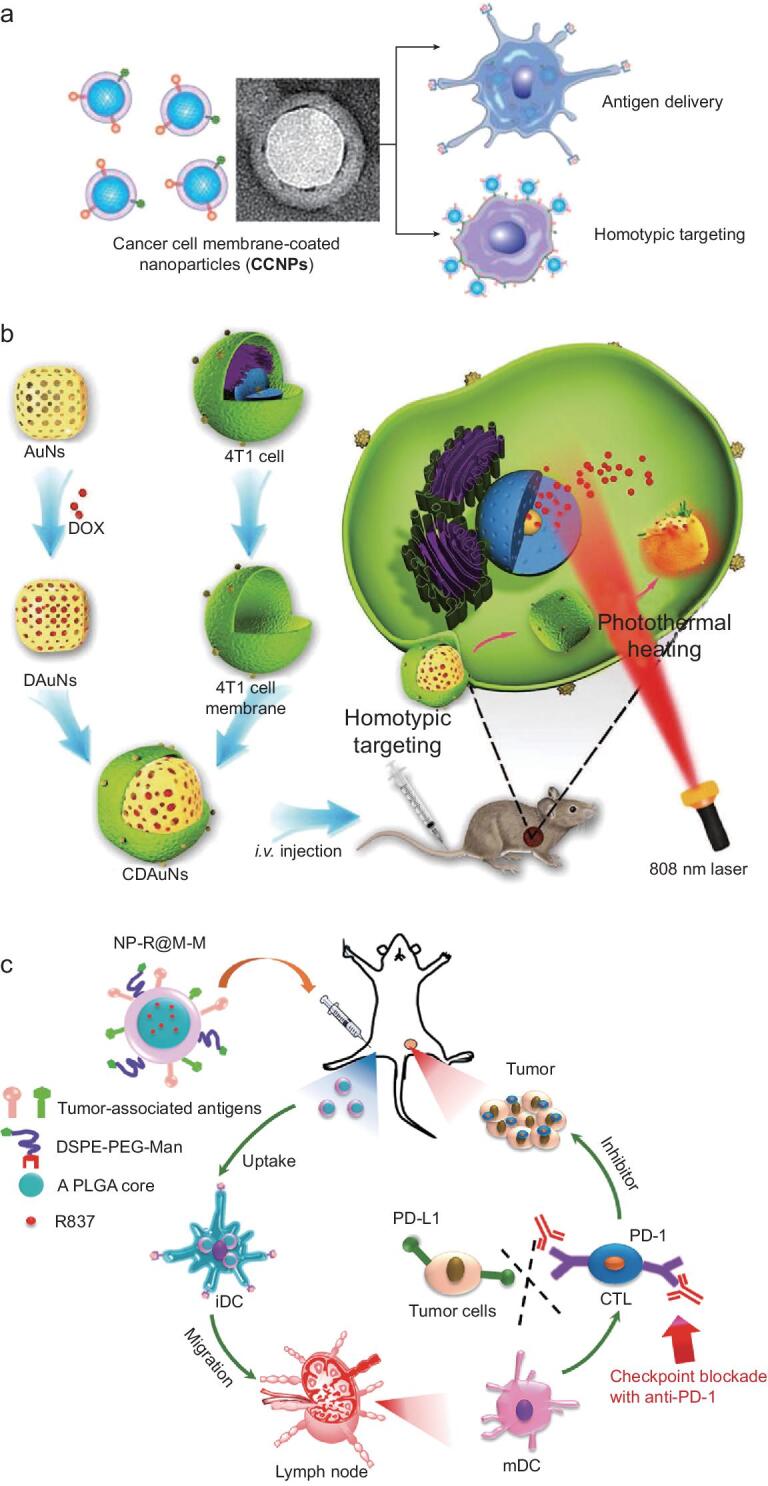
Schematic of the cancer cell membrane-coated nanoparticles. (a) Cancer cell membranes with associated antigens are coated on the surface of PLGA nanoparticles for anticancer vaccination and drug delivery [90], Copyright 2014, American Chemical Society. (b) Cancer cell membrane-coated gold nanocages used for hyperthermia-induced drug release to inhibit the growth and metastasis of cancer cells [92], Copyright 2015, Wiley-VCH. (c) Anticancer vaccine composed of cancer cell membrane-coated nanoparticles [93], Copyright 2018, American Chemical Society.

The exploitation of new sources of cells for specific functions and biomedical purposes is continuously reported. Conventional cells without canceration, including RBCs and immune cells, have been used for biomimicking synthetic nanosystems. However, the unexpected result is that cancer cell membranes have also attracted the interests of researchers for use as the coating materials for nanoparticles. Cancer cell membranes possess a variety of unique features, including a limitless replicative potential, resistance to cell death, immune escape, a long circulation time, and homologous binding capabilities; these properties make them a novel type of coating material for designing anticancer therapeutic systems [88,89]. Moreover, membrane-related tumour antigens and specific functional proteins are of great importance and must be considered when constructing anticancer vaccines. The derivation of cancer cell membranes to cover synthetic nanosystems has inspired a new method to develop promising therapeutic platforms. Cancer cell membranes are always derived using the hypotonic lysis method. Cancer cell membrane-coated PLGA nanoparticles not only represent an effective approach to introduce membrane-bound antigens that promote a tumour-specific immune response and binding process but also represent a new method of drug delivery, which demonstrates a perspective of cancer therapy from drug delivery to immunotherapy (Fig. 4a) [90].

Due to the antigenic diversity of membranes, cancer cell membrane-coated nanoparticles are expected to be promising and useful tools for biomedical applications. A dual-modal theranostic nanoplatform is fabricated by cloaking cancer cell membranes on indocyanine-green-loaded lipid-polymer nanoparticles, endowing them with the functionalization of the homologous binding capability, improved cell endocytosis, accumulation in the target tumour, imaging and photothermal therapy [91]. Researchers have further used different kinds of nanoparticles as cores coated with cancer cell membranes. A biomimetic system composed of gold nanocages with a cancer cell membrane coating has been used for drug delivery (DOX) and exhibited an enhanced permeability and retention (EPR) effect and high levels of accumulation in the tumour area (Fig. 3b). Following irradiation with near-infrared light, this biomimetic system induces hyperthermia-triggered drug release to suppress the growth and metastasis of breast cancer [92]. Recently, immunotherapy has attracted tremendous interest due to the award of a Nobel Prize in this area in 2018. An anticancer vaccine composed of receptor 7 agonist (imiquimod) loaded-PLGA nanoparticles coated with mannose-modified cancer cell membranes enhances the uptake by dendritic cells to trigger the immune response toward cancer cells and subsequently delay tumour development (Fig. 3c) [93].

An oxygen self-sufficient platform based on the cancer cell membrane and a porous zeolitic imidazolate framework encapsulated with Al(III) phthalocyanine chloride tetrasulfonic acid and catalase has been designed for oxygen-dependent photodynamic therapy [94]. In addition to supplying oxygen, cancer cell membrane-based biomimetic nanoparticles could be used as sensors to monitor low oxygen levels in the tumour region. Moreover, a porphyrinic metal–organic framework consisting of Pt(II) meso-tetra(4-carboxyphenyl)porphyrin and Zr_6_ clusters covered with cancer cell membranes has been used for tumour targeting and phosphorescence image-guided photodynamic therapy. This system exhibited great performance in oxygen sensing, homotypic targeting, and immune cell evasion, as well as a desirable efficacy of photodynamic therapy [95]. For the treatment of metastatic cancer, a nanosystem composed of a PTX-loaded polymeric nanoparticle with a cancer cell membrane coating displays higher accumulation in tumours, indicating that specific targeting of homotypic tumours is achieved. In addition, cancer cell membrane-coated nanoparticles are responsible for both the targeted delivery and low internalization of macrophages and are regarded as a smart platform for the treatment of homotypic metastatic cancers [96,97]. Overall, the emerging cancer cell membrane-coated therapeutic systems represent a safe and effective strategy for cancer immunotherapy and significantly bridge the gap between tumour cell-based materials and cancer therapy, while holding great potential for biomedical applications.

### Bacterial membrane-coated nanoparticles

The use of bacteria as a biohybrid therapeutic system has a long history [98]. Like cell-based biomimetic systems, the protein shells of bacteria conjugate with functional nanoparticles and antibodies for drug delivery, tumour imaging, long retention, and cancer therapy [99]. In particular, bacterium-based systems survive in anaerobic environments and along oxygen taxis to reach anoxic tumours *in vivo*. For example, *Bifidobacterium breve* and *Clostridium difficile* mediated delivery systems use their anaerobic habitats to target hypoxia tumour tissues for imaging and therapy [100,101]. Based on the aforementioned great properties of bacterium-mediated nanosystems, the concept of a cell membrane-camouflaged strategy has been further considered to use ‘particular cell membranes’ harvested from bacteria. Compared with cell membrane-coated nanosystems, the bacterial membrane-coating strategy presents a new perspective from design to the construction of the biomimetic platforms for biomedical applications. A large number of bacteria have evolved high affinities for specific mammalian cells and tissues via their ligands. Some bacteria naturally possess tumour-targeting features mediated by adhesion proteins, antigens, or other molecules on the surface of the protein shell. In early studies, bacterial ghosts were derived and further designed as advanced targeting delivery carriers for the targeted delivery of drugs and RNA [102]. Therefore, like mammalian cell membranes, the various properties of bacterial membranes represent a promising topic to explore and develop them as coating materials for synthetic nanoparticles.

**Figure 5.**
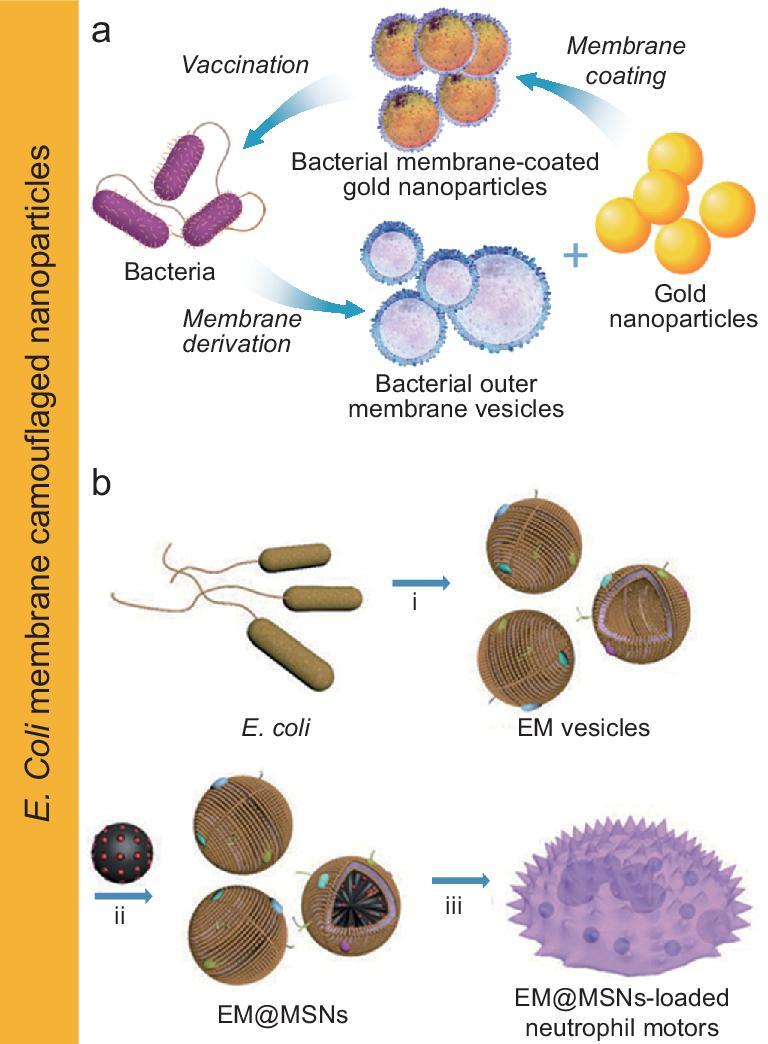
Schematic of bacterial membrane-coated nanoparticles. (a) Biointerfacing gold nanoparticles with *E. coli* membranes for modulating antibacterial immunity [103], Copyright 2015, American Chemical Society. (b) MSNs coated with *E. coli* membranes for the construction of a neutrophil-based active drug-delivery system [104], Copyright 2017, Wiley-VCH.

Although fewer researchers have investigated bacterial membrane-coated nanoparticles, these platforms are still part of the family of cell membrane-covered systems. Unlike the isolation of mammalian cell membranes using hypotonic lysis, the isolation of gram-negative bacteria (*Escherichia coli* (*E. coli*)) outer membranes is achieved by gradient ultracentrifugal filtration [103]. After removing the genetic material inside the bacteria, researchers harvest the outer membrane of *E. coli* and translate it into vesicles for reconstitution on the surface of gold nanoparticles (AuNPs). These resulting core–shell particles exhibit remarkable stability in physiological environments and membrane-bound immunogenic antigens with intrinsic adjuvant features that enable core nanoparticles to serve as antibacterial vaccines that promote an adaptive immune response against bacterial invasion. Additionally, bacterial membranes have been coated on MSNs to design biohybrid drug-delivery systems [104]. After coating with *E. coli* membranes, large quantities of DOX-loaded MSNs are engulfed by neutrophils due to the immune reaction, and this biohybrid drug-delivery system with natural immunological recognition has been successfully developed (Fig. 5b). This delivery system employs the intrinsic chemotaxis capability of neutrophils to distribute the cargo payload, resulting in controllable movement along the gradient of chemical signals generated by the targeting *E. coli* for targeted drug delivery.

Overall, the bacterial membrane-coating approach represents a new and effective surface-engineering method for nanoparticles instead of directly using bacteria. This biomimetic strategy not only simplifies the complexity of current bacterium-based drug-delivery systems, but also miniaturizes this biohybrid nanosystem into the nanoscale. From the perspective of biological safety, the bacterial membrane-coating strategy is biocompatible with living organisms because an emptied bacteria ghost is used that is quite safe for cancer therapy, which challenges the concept of conventional biological safety. Currently, bacterial membrane coatings are still in the optimization stage and have not yet achieved the versatility of the regular cell membrane-coating process; thus, this field requires substantially more effort.

## CONCLUSIONS AND PERSPECTIVES

We have reviewed the state-of-the-art of cell membrane-covered nanoparticles and highlighted relevant biomedical applications. The biomimetic strategy aims to fabricate cell-like nanoparticles through a top-down assembly approach that innovatively bridges the gap between synthetic materials and biological entities, further inspiring the new design of theranostic systems from the biological perspective. The proposed technique also represents a chemistry-free surface-engineering approach for nanoparticles that incorporates the bioactivity of membrane-related components. The specific functions and properties of the cell membrane, such as long circulation time, immune escape, antibioadhesion, and tissue-specific targeting are transferred to the synthetic materials. Meanwhile, the extraordinary diversity of parent cells (RBCs, platelets, stem cells, cancer cells, immune cells, and bacteria) provides a wealth of coating types to conjugate with nanoparticles for various biomedical applications. Additionally, most synthetic nanoparticles can be covered by cell membranes, regardless of their hydrophobicity, surface potential, size, and morphology, leading to the formation of biomimetic platforms with biocompatibility and a lack of immunogenicity.

In future applications, cell membrane-covered nanoparticles will show a number of advantages in the aspects of *in vivo* drug delivery, bioimaging, and cancer therapy. However, the use of a single type or conventional types of cell membranes as the coating materials may limit the diversity of functions. Mixed types of cell membranes or new types of cell or bacterial membranes should be employed as coating components in the future. By introducing comprehensive biological moieties and functions, many integrated functions will enhance the *in vivo* performance of therapeutic nanoparticles. Facile ligands composed of antibodies, DNA/RNA, peptides, proteins, and enzymes should be considered for incorporation into cell membrane coatings to enhance the synergistic performance in biomedical applications. Furthermore, cell membrane-covered synthetic nanoparticles are still limited by complex preparation methods, easy deactivation, large-scale synthesis, and difficult preservation; these should be investigated further and addressed in the future. Additionally, researchers examining cell membrane-covered nanoparticles should focus on their translation from experimental research to clinical applications.
